# Meta-analytical methods for estimating outcomes from overall response rate in patients with relapsed/refractory diffuse large B-cell lymphoma

**DOI:** 10.18632/oncotarget.26904

**Published:** 2019-05-14

**Authors:** Ling Wang, Hung Lam, Yaping Shou, Aaron Galaznik

**Affiliations:** ^1^ Millennium Pharmaceuticals, Inc., a wholly owned subsidiary of Takeda Pharmaceutical Company Limited, Cambridge, MA, USA; ^2^ MMS Holdings Inc., Canton, MI, USA; ^3^ Currently employed at Pfizer, Inc.; ^4^ Currently employed at Trillium Therapeutics Inc.; ^5^ Currently employed at SHYFT Analytics, a Medidata Company

**Keywords:** diffuse large B-cell lymphoma, meta-analyses, outcomes, overall response rate, durable response rate

## Abstract

Relapsed/refractory diffuse large B-cell lymphoma (DLBCL) is highly heterogeneous and current trials are investigating new approaches to improve outcomes. Limited data on response endpoints can confound estimation of a treatment effect when designing studies of novel agents in this setting, which can hinder study sample size calculations, especially if a net estimate is required for a ‘physician’s choice’ comparator arm. Here we estimate complete response rate (CRR), overall response rate (ORR), and extrapolate durable response rates (DRR; CR/partial response lasting ≥16 weeks) for such a comparator arm from published ORRs in DLBCL.

CRR, ORR, and DRR (if reported) were obtained from published clinical trials for approved single-agent therapies in patients with relapsed/refractory aggressive non-Hodgkin lymphoma after ≥2 prior therapies. Meta-analyses were performed to estimate CRR, ORR, and DRR based on ORR data reported from these studies.

Published data from studies of eight monotherapies were included. Meta-analyses using fixed and random effects models showed a pooled estimate for a CRR of 12% (95% confidence interval [CI]: 9−15) and 11% (95% CI: 8−15), respectively, an ORR of 30% (95% CI: 25−35) and 30% (95% CI: 24−36), respectively, and a DRR of 14% (95% CI: 11−18; same for fixed and random effects models). Bayesian meta-analysis estimated a pooled DRR of 14% (95% credible interval: 11−19).

CRR estimates for a physician’s choice comparator arm in patients with relapsed/refractory DLBCL were 11−12%; DRR estimates were 14% regardless of methodology. Lack of consistency in reported data and choice of endpoints can be addressed using meta-analytic approaches.

## INTRODUCTION

Diffuse large B-cell lymphoma (DLBCL) is an aggressive form of non-Hodgkin lymphoma (NHL) representing approximately 30% of all NHLs, making it the most common subtype [1–3]. The current standard of care for frontline treatment of DLBCL is rituximab in combination with cyclophosphamide, doxorubicin, vincristine, and prednisone (R-CHOP) [3, 4]. Compared with regimens not containing rituximab, R-CHOP offers potentially curative therapy with significant survival benefits [5–7]; however, a substantial proportion of patients have disease that either is refractory to or relapses after initial therapy [3, 4, 8]. Patients who relapse following initial treatment but remain chemosensitive are typically treated with high-dose chemotherapy followed by autologous stem cell transplantation (ASCT) [9–11]. For those who do not respond to salvage regimens or have poor outcomes post-ASCT, there is an unmet need for treatment of third-line relapsed or refractory (R/R) DLBCL, as currently there are no approved drugs and no standard of care in this setting [8, 12, 13].

The R/R DLBCL treatment landscape is rapidly evolving, with multiple new treatments currently under investigation. Novel agents or regimens need to be investigated in the context of this complex clinical landscape, with clinically relevant endpoints required to demonstrate superiority against existing agents. In R/R DLBCL, the published data on response endpoints are limited, and there is a lack of consistency in the data reported and the choice of efficacy endpoints in single-agent studies. This makes it difficult to estimate standard-of-care response rates and can confound estimation of a threshold or comparator rate treatment effect when designing studies of novel agents in this setting. In turn, this hinders study sample size calculations, which are needed to inform clinical trial design.

Overall response rate (ORR) or complete response rate (CRR) has been widely used as the primary endpoint in studies in R/R DLBCL. However, durable response rate (DRR), defined as the percentage of patients with a time interval of ≥16 weeks from first CR or partial response (PR) to progression of disease or death, may be a more clinically relevant endpoint as it captures both the proportion of patients achieving a response and the duration of these responses in a single endpoint. Furthermore, the US Food and Drug Administration guidance for Approval of Cancer Drugs and Biologics indicates the importance of durability of clinical response for assessing clinical benefit in oncology therapies, particularly when overall survival (OS) data are not yet available [14, 15]. However, to date, the only reported use of DRR in DLBCL was in the EXTEND study of pixantrone versus single-agent chemotherapies, in which 17.1% of pixantrone-treated patients achieved a DRR [16]. In a limited study of vorinostat in R/R DLBCL, the single responder among the 18 enrolled patients had a durable response lasting for at least 4 months, equating to a DRR of 6% [17].

The purpose of this paper is to illustrate a methodological approach for addressing this gap in available response durability data in R/R DLBCL. Starting with a targeted literature review, we used meta-analytic approaches to estimate CRR and ORR as well as to extrapolate DRR from reported ORR data for a physician’s choice comparator arm of single-agent therapy against which to compare a novel agent. Results can be used to inform the design of future clinical trials in this setting.

## RESULTS

### Selection of studies

Following our targeted literature review, we identified 10 studies of eight monotherapies that were eligible for inclusion in the present analyses [16–25]. ORR was reported in eight studies and DRR was reported in two studies. The key patient characteristics, study details, and response rates reported in these studies are summarized in Table 1.

**Table 1:** Studies of single-agent therapies identified in our targeted literature review and the reported data on ORR, CRR, and DRR

Regimen	Patients, *n*	Median age, years (range)	Study design	Median number of prior therapies	ORR (%)	CR (%)	Reported DRR (%)
Rituximab [18]	54	62.5 (20–83) Group A 65 (32–86) Group B	Phase II, open-label, randomized	NR	31	9	-
Gemcitabine [20]	30	61	Phase II	2	20	0	-
Bendamustine [22]	18	66 (38–84)	Phase II	2	44	17	-
Oxaliplatin [21]	22^a^	62 (28–79)^b^	Phase II	2^b^	32^a^	9^a^	-
Vorinostat [17]	18	66.5 (59–86)	Phase II	2	6	6	6
Pixantrone [16]	70^c^	60 (18–80)	Phase III, open-label, randomized	3	37	20	17
Ibrutinib [24]	80	60 (34–89) ABC 65 (28–92) GCB 63 (44–85) Unclassified 65 (58–78) Unknown	Phase I/II	3^d^ 3.5^f^	25^e^	10	-
Lenalidomide [19] (for DRR estimation)	51^g^	69 (28–84)	Phase II/III, open-label, randomized	2	28	10	-
Lenalidomide [23] (for CRR estimation)	49 (*n* = 26 for DLBCL only)	65 (23–86)	Phase II	4	35 (19 for DLBCL)	12 (12 for DLBCL)	-
Lenalidomide [25] (for CRR estimation)	217 (*n* = 108 for DLBCL)	66 (21–87)	Phase II	3	35 (28 for DLBCL)	13 (7 for DLBCL)	-

^a^Subset of patients with aggressive NHL; ^b^Based on total number of patients (*N* = 30); ^c^Number of patients in the pixantrone arm (total study *N* = 140); ^d^Based on patients classified as ABC, unclassified, and unknown; ^e^ORR = 37% in a subset of patients with activated B-Cell DLBCL; ^f^Based on patients classified as GCB; ^g^Number of patients in the lenalidomide arm (total study *N* = 102).

Abbreviations: ABC, activated B cell-like; CR, complete response; CRR, complete response rate; DLBCL, diffuse large B-cell lymphoma; DRR, durable response rate; GCB, germinal center B cell-like; NR, not reported; ORR, overall response rate.

### Frequentist meta-analysis

**Figure 1:**
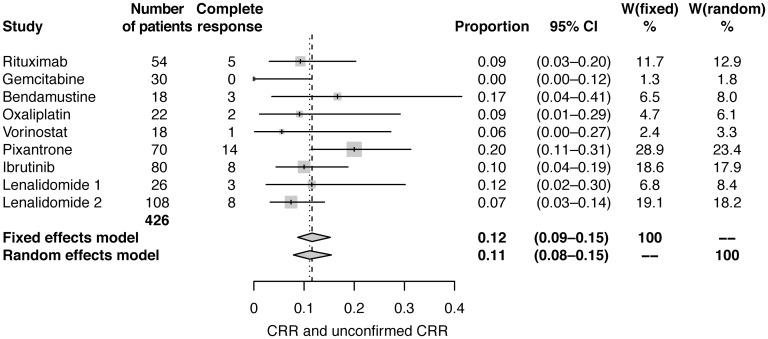
Forest plot of frequentist meta-analysis estimated CRRs. Columns W(fixed) and W(random) are the weights used in the fixed effect and random effect models [26].

**Figure 2:**
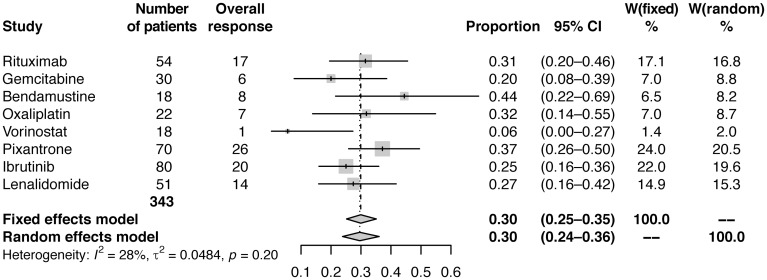
Forest plot of frequentist meta-analysis estimated ORRs. Columns W(fixed) and W(random) are the weights used in the fixed effect and random effect models [26].

Fixed and random effect analyses were conducted using standard meta-analysis practice, including calculation and application of weights for each study [26]. Meta-analysis using fixed effects and random effects modelling showed pooled estimates of CRR of 12% (95% CI: 9–15) and 11% (95% CI: 8–15), respectively (Table 2; Figure 1), and estimates of ORR of 30% (95% CI: 25−35) and 30% (95% CI: 24−36), respectively (Table 2; Figure 2).

**Table 2:** Pooled and individual estimated CRRs (frequentist meta-analysis), ORRs (frequentist meta-analysis), and DRRs (frequentist and Bayesian meta-analysis) for the included studies

Regimen	Frequentist meta-analysis			Bayesian meta-analysis
	CRR (95% CI)	ORR (95% CI)	DRR (95% CI)	DRR (95% CI)
Rituximab [18]	0.09 (0.03–0.20)	0.31 (0.20–0.46)	0.15 (0.07–0.27)	0.161
Gemcitabine [20]	0.00 (0.00–0.12)	0.20 (0.08–0.39)	0.10 (0.02–0.27)	0.1092
Bendamustine [22]	0.17 (0.04–0.41)	0.44 (0.22–0.69)	0.22 (0.06–0.48)	0.2249
Oxaliplatin [21]	0.09 (0.01–0.29)	0.32 (0.14–0.55)	0.14 (0.03–0.35)	0.1666
Vorinostat [17]	0.06 (0.00–0.27)	0.06 (0.00–0.27)	0.06 (0.00–0.27)	0.04996
Pixantrone [16]	0.20 (0.11–0.31)	0.37 (0.26–0.50)	0.17 (0.09–0.28)	0.1874
Ibrutinib [24]	0.10 (0.04–0.19)	0.25 (0.16–0.36)	0.11 (0.05–0.20)	0.1279
Lenalidomide	0.12 (0.02–0.30) [23] 0.07 (0.03–0.14) [25]	0.27 (0.16–0.42) [19]	0.14 (0.06–0.26) [19]	0.1413
Pooled results	Fixed: 0.12 (0.09–0.15) Random: 0.11 (0.08–0.15)	Fixed: 0.30 (0.25–0.35) Random: 0.30 (0.24–0.36)	Fixed: 0.14 (0.11–0.18) Random: 0.14 (0.11–0.18)	0.1443 (0.1053–0.1873)

Abbreviations: CI, confidence interval; CRR, complete response rate; DRR, durable response rate; ORR, overall response rate.

**Figure 3:**
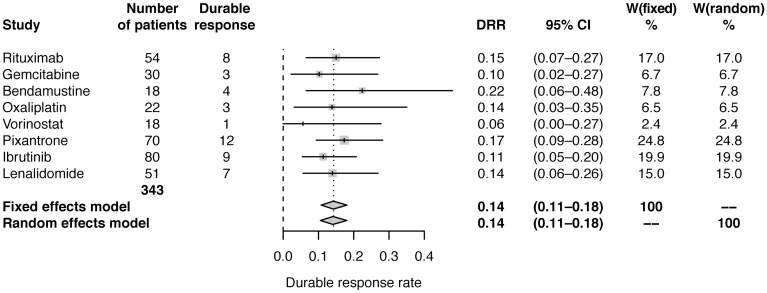
Forest plot of frequentist meta-analysis estimated DRRs. Columns W(fixed) and W(random) are the weights used in the fixed effect and random effect models [26].

Using data from studies of pixantrone and vorinostat [16, 17] and assuming a simple relationship between ORR and DRR of DRR=r*ORR, the correlation coefficient was estimated to be r=0.4696. This relationship was used to estimate DRR in the studies of the other six agents. Estimated DRR was 14% (95% CI: 11–18) modelled with both fixed effects and random effects (Table 2; Figure 3).

### Bayesian meta-analysis

**Figure 4:**
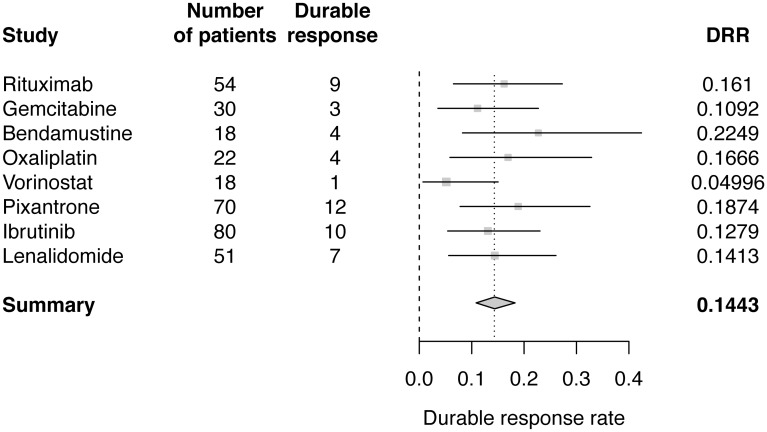
Forest plot of Bayesian meta-analysis estimated DRRs.

A separate Bayesian meta-analysis was performed using published results of ORR and DRR for the eight monotherapies. Posterior distribution of the ratio between ORR and DRR was estimated using data from studies of pixantrone and vorinostat [16, 17]. DRRs for the other six monotherapies were then estimated. Estimated value for r was 0.4758 (95% CI: 0.2391–0.8119). Bayesian meta-analysis estimated the pooled DRR, assuming a normal distribution for the log odds of DRR, to be 14.43% (95% CI: 10.53–18.7) (Table 2; Figure 4). The Bayesian estimate of the pooled DRR was very similar to results from the frequentist meta-analysis approach using both fixed and random effects models.

## DISCUSSION

Estimating effect size is a crucial aspect of sample size calculation for any clinical trial. For trials comparing to the standard of care, whether in the form of a comparator arm or a historic benchmark, this is challenging when the standard-of-care treatment options are highly fragmented and rapidly evolving. In DLBCL, treatment guidelines include many potential regimens for second-line and subsequent therapy [3, 27, 28] and real-world treatment patterns are heterogeneous [29]. However, there is no established standard of care for third-line or later therapy in DLBCL. Furthermore, published studies may not include all endpoints of interest, as seen for DRR in R/R DLBCL. These trends make estimation of effect size, especially in a comparator population receiving a variety of treatments, difficult to assess.

Here we outline a meta-analytical process both for estimation of anticipated standard-of-care response rates in terms of ORR and CRR, as well as for extrapolation to anticipated DRR when information for this endpoint is not available. This methodology provides a quantitative approach to extrapolation using multiple sources. In addition to providing estimates, it also allows for a statistically rigorous approach to quantifying the uncertainty around those estimates. Furthermore, this approach can be implemented quickly, using publicly available clinical trial results. Given the known challenges with sharing of clinical trial data, particularly across multiple sponsors, this enables faster generation of estimates for clinical study planning [30].

In this study, two frequentist and one Bayesian meta-analysis methods were used to estimate response rates and extrapolate DRRs based on published ORRs from various studies investigating single-agent regimens in patients with R/R DLBCL. In frequentist meta-analysis using fixed and random effects modelling, pooled estimates of CRR were 12% and 11% and pooled estimates of ORR were 30% and 30%, respectively. The pooled DRR estimates were very similar across analyses, with an estimated pooled DRR of 14% using fixed effects and random effects modelling, and 14.4% predicted using Bayesian meta-analysis. The consistency of the predicted DRRs across different methods demonstrates the robustness of the final estimate.

The benefit of calculating these estimates is twofold. First, by estimating the likely CRR and ORR rates and ranges for different agents, one can quantitatively estimate the likelihood of different effect sizes of a new therapeutic against different potential comparators. This can thereby inform sample size decisions in the backdrop of a desired power to detect a given magnitude of effect [31]. Second, for this particular example, this approach allows extrapolation of potential DRR rates and quantifying the uncertainty around them, which is important given the paucity of available information for this measure.

The largest limitation of the present meta-analysis is the assumption made for the extrapolation of DRR from ORR, as this is based on data reported from only two studies. Furthermore, there is heterogeneity between the studies in terms of patient characteristics, study designs, and patient numbers. In addition, the studies included in this meta-analysis were restricted to those of monotherapies only; however, combination regimens are also commonplace in the R/R DLBCL setting.

Future enhancements to the current analysis would include use of a systematic literature review approach for study identification, following Cochrane or Centre for Reviews and Dissemination York guidelines, as well as expansion to include combination regimens. Given the lack of treatment guidelines for later-line DLBCL, regimen selection could be done in consultation with clinical oncology experts. Other endpoints, such as progression-free survival (PFS) or OS could also be evaluated.

The approach outlined in this study also illustrates how meta-analytical techniques may be used to overcome common issues with estimating an effect size for a threshold efficacy target or for a physician’s choice comparator arm in studies of patients with R/R DLBCL, including inconsistent types of data reported and choice of endpoints. These data can be used to inform efficacy targets (DRR, ORR, or CRR) as well as sample size calculations in future clinical trials investigating new treatments in R/R DLBCL. Consequently, meta-analytical techniques should be considered when estimating standard-of-care treatment effects in other disease areas similarly characterized by fragmented, evolving treatment landscapes and inconsistent capture of desired endpoints.

## MATERIALS AND METHODS

### Targeted literature review

We conducted a targeted literature review of PubMed from December 1998 to December 2016 to capture publications including and subsequent to early-phase studies of rituximab and gemcitabine [18, 20]. Search parameters included terms pertaining to aggressive NHLs and assessment of efficacy (ORR, CRR, DRR). Studies of monotherapies were included if they reported efficacy results (at least ORR and CRR) in a R/R (≥2 prior lines of therapy) DLBCL population. Studies of combination regimens or not reporting efficacy results specific to a DLBCL population were excluded.

### Data collection and statistical analysis

CRR, ORR, and DRR (where reported) for patients with R/R DLBCL were obtained from selected studies. Two meta-analytical approaches were used to estimate response rates and extrapolate DRR from the reported data. A frequentist meta-analysis approach used fixed effects and random effects meta-analysis models to obtain an overall estimate of response from the data reported on existing DLBCL treatments. The ORR and DRR reported for the existing treatments for DLBCL (Table 1) showed a relationship between ORR and DRR for vorinostat and pixantrone. Data from these two drugs were used to estimate the relationship between ORR and DRR. The ranges of distribution for both ORR and DRR were between 0 and 1, and all durable responses were responses such that DRR≤ORR. Therefore, it was reasonable to assume that DRR=r*ORR, where 0<r≤1. A least squares approach using data from vorinostat and pixantrone studies was used to estimate r, which was then used to estimate DRR in other studies. The number of durable responders was then calculated using the sample size for each study.

Meta-analysis on the estimated DRRs using fixed and random effects models [32] was used to estimate pooled DRR. A Bayesian meta-analysis [33] was carried out using the published values for ORR and the available values for DRR. The ratio of DRR and ORR was estimated by assuming beta distributions for ORR and DRR, and binomial distributions for the number of responders and number of durable responders: n_resp_~binomial(ORR, n), ORR~Beta(a_1_, a_2_), n_duresp_~binomial(DRR, n), DRR~Beta(b_1_, b_2_), r=DRR/ORR, with hyper parameters a_1_, a_2_, b_1_, b_2_ all following inverse gamma distribution IG(0.001, 0.001). The posterior distribution of r was approximated by a Beta distribution given that 0<r≤1. Using a grid search of the Beta (a, b) distribution parameters a and b, with the search range between 0 and 10 by 0.01, we found the optimal a and b by minimizing the sum of the square distances of the 2.5th, 50th, 97.5th percentiles between each of the tried Beta distribution and the posterior quantiles of r. The Beta distribution identified using this method was Beta (5.06, 5.07). From this, ratio~Beta(5.06, 5.07), ORR~Beta(1, 1), n_resp_~binomial(ORR, n), n_duresp_=r*n*ORR estimated the number of durable responses for each study. Finally, the meta-analysis was performed by estimating the posterior mean of logit(DRR), which was assumed to be normally distributed: Normal distribution: n_duresp_~binomial(DRR, n), logit(DRR)~Normal(μ, 1/*τ*) with hyperparameters μ~Normal(0, 1/0.0001), *τ* ~InverseGamma(0.001, 0.001). Overall DRR for the meta-analysis was calculated by e^μ^/(1+ e^μ^).

## Abbreviations

ASCT: autologous stem cell transplantation
CR: complete response
CRR: complete response rate
DLBCL: diffuse large B-cell lymphoma
DRR: durable response rate
NHL: non-Hodgkin lymphoma
ORR: overall response rate
OS: overall survival
PFS: progression-free survival
PR: partial response
R/R: relapsed/refractory
R-CHOP: rituximab in combination with cyclophosphamide, doxorubicin, vincristine, and prednisone
